# Fighting Obesity-Related Micronutrient Deficiencies through Biofortification of Agri-Food Crops with Sustainable Fertilization Practices

**DOI:** 10.3390/plants11243477

**Published:** 2022-12-12

**Authors:** Carlos Esteban Guardiola-Márquez, María Teresa Santos-Ramírez, M. Eugenia Segura-Jiménez, Melina Lizeth Figueroa-Montes, Daniel A. Jacobo-Velázquez

**Affiliations:** 1 Tecnologico de Monterrey, Escuela de Ingenieria y Ciencias, Ave. General Ramon Corona 2514, Zapopan 45138, Jalisco, Mexico; 2 Tecnologico de Monterrey, The Institute for Obesity Research, Ave. General Ramon Corona 2514, Zapopan 45201, Jalisco, Mexico

**Keywords:** agri-food systems, beneficial soil microorganisms, biofertilization, food biofortification, malnutrition, micronutrients, nanofertilization, nanoparticles, obesity

## Abstract

Obesity is a critical medical condition worldwide that is increasingly involved with nutritional derangements associated with micronutrient deficiencies, including iron, zinc, calcium, magnesium, selenium, and vitamins A, C, D, and E. Nutritional deficiencies in obesity are mainly caused by poor-quality diets, higher nutrient requirements, alterations in micronutrient metabolism, and invasive obesity treatments. The current conventional agricultural system is designed for intensive food production, focusing on food quantity rather than food quality, consuming excessive agricultural inputs, and producing nutrient-deficient foods, thus generating severe health and environmental problems; agricultural food products may worsen obesity-related malnutrition. Therefore, modern agriculture is adopting new biofortification technologies to combat micronutrient deficiencies and improve agricultural productivity and sustainability. Biofertilization and nanofertilization practices are increasingly used due to their efficiency, safety, and reduced environmental impact. Biofertilizers are preparations of PGP-microorganisms that promote plant growth by influencing plant metabolism and improving the nutrient uptake, and nanofertilizers consist of synthesized nanoparticles with unique physicochemical properties that are capable of increasing plant nutrition and enriching agricultural products. This review presents the current micronutrient deficiencies associated with obesity, the modern unsustainable agri-food system contributing to obesity progression, and the development of bio- and nanofertilizers capable of biofortifying agri-food crops with micronutrients commonly deficient in patients with obesity.

## 1. Introduction

Obesity is a highly prevalent chronic medical condition characterized by the excessive or abnormal accumulation of body fat (adiposity) resulting from an imbalance between the energy consumed and the energy expended [1,2,3,4,5,6]. It is defined with a body mass index (BMI), estimated as weight/height^2^ (kg/m^2^), of 30 kg/m^2^ or above, and is associated with serious negative implications on human health and quality of life; in particular, excess body fat has been shown to negatively affect the metabolism of micronutrients in obese patients. In addition, obese individuals are susceptible to nutritional derangements because their diet is mainly based on inexpensive, energy-dense, and low-micronutrient quality foods [2,7,8]. Obese people are now facing a complex nutritional challenge characterized by the coexistence of under- and overnutrition. This concept has been recently defined as a “double burden of malnutrition”, involving an excessive consumption of calories associated with a shortage of certain microelements [5,9,10]. Several studies have reported a direct and clear link between obesity and various micronutrient deficiencies, including iron, zinc, magnesium, potassium, selenium, and vitamins A, C, E, and D [2,6,11,12,13]. These deficiencies can aggravate the obese phenotype and promote the development of comorbidities. For instance, vitamin A and C inadequacies correlate with leptin concentrations and elevated adipogenesis and fat deposition [2,13,14].

In addition to bad dietary choices and alterations in the metabolism of nutrients as causes of obesity-related micronutrient deficiencies, insufficient access to nutrient-rich foods, which is closely related to modern agricultural practices and the current agri-food system, also contributes significantly to the prevalence of these conditions in obese subjects [6,9,14]. However, modern agriculture faces critical challenges in solving health and environmental issues associated with macro and micronutrient deficiencies, food insecurity, low fertilizer-use efficiency, overfertilization, climate change, water scarcity, a reduction in agricultural lands, and soil degradation [15,16,17,18,19,20,21]. Long-term effects of modern intensive agronomic practices include significant losses in crop productivity and the nutritional value of agricultural products [17,20,21,22,23,24,25].

A sustainable food system provides sufficient, safe, nutritious, accessible, and affordable food to meet current dietary needs while preserving healthy environments and ecosystems that can supply future generations with a minimal negative environmental impact [26,27]. The agri-food system needs a significant transformation to be environmentally sustainable and productive [27,28]. Therefore, modern agriculture is adopting new biofortification technologies through fertilization to combat human micronutrient deficiencies and improve agricultural productivity and sustainability [19,29,30,31].

Firstly, the use of biofertilizers based on plant-growth-promoting microorganisms (PGPM) is a promising strategy for enhancing plant growth and food quality without environmental contamination; PGPM mobilizes soil nutrients, improves macro- and micronutrient bioavailability, produces plant growth regulators, protects crops from phytopathogens, and improves the soil structure [25]. Microorganism-mediated improvements in plant development are a relevant strategy for promoting the sustainability of the current agri-food system [15,17,20,32]. Research should focus on studying native microbial species as these have exhibited more significant benefits on plant growth promotion than non-native or commercial strains [33,34]. The other strategy is nanofertilization, which consists of applying nanosized minerals to facilitate the uptake and assimilation of nutrients by the crops, enhance plant nutrition, and reduce chemical fertilizer consumption and nutrient-related toxicity [35]. Both methods have been successfully applied to biofortify plants with mineral elements, vitamins, and other bioactive compounds [30].

The manuscript aims to correlate agriculture with a challenging global health problem: obesity-related micronutrient deficiencies. To achieve this, we present a detailed overview of the most frequent micronutrient deficiencies associated with obesity, its leading causes, and its health implications, followed by the current unsustainable agri-food system contributing to an increase in the prevalence of nutritional inadequacies, and, finally, the biofertilization and nanofertilization strategies based on plant-growth-promoting microorganisms and micronutrient nanoparticles capable of biofortifying agri-food crops with micronutrients commonly deficient in patients with obesity. This review also includes some important considerations for the formulation and proper use of these fertilization practices, such as the use of native consortia of soil microorganisms and biosynthesized nanoparticles. The information in the manuscript comes from several journals and databases, researching and incorporating the most relevant studies of the last five years. We attempt to improve the understanding of how these sustainable fertilization practices can be applied to fight nutritional deficiencies of global relevance, as well as reinforce the relevance and urgency of taking sustainable actions in the agricultural sector since it not only exerts a harmful impact on the environment, but also affects many aspects of people’s health and quality of life.

## 2. Nutritional Deficiencies Associated with Overweight or Obese Patients

Obesity is a nutritional imbalance that negatively alters the micronutrient status of individuals; it has been recognized as a crucial risk factor for various nutrient deficiencies, being increasingly associated with an inadequate intake of minerals such as iron, calcium, magnesium, zinc, and copper, as well as vitamins (folate, vitamin A, D, and B_12_) [2,6,14,36]. Most micronutrients act as cofactors for the functioning of enzymes in living organisms and therefore regulate many vital metabolic processes in the body [6,23]. Deficiencies or a lack of homeostasis of micronutrients can cause severe implications for human health, such as congenital disabilities, stunted growth, learning disabilities, immune dysfunction, cancer, cardiovascular disease, defective antioxidant defense mechanisms, osteoporosis, neurodegenerative disorders, intestinal microbiota malfunction, deteriorates the functionality of most organs and systems, and contributes to the aggravation of many diseases. Since micronutrients are implicated in fat and carbohydrate metabolism, glucose metabolic pathways, the insulin-signaling cascade, and pancreatic β-cell function, their deficiency worsens the development of obesity [2,10,14,23,24].

A poor diet quality mainly causes the occurrence of nutritional deficiencies in obesity based on the overconsumption of processed foods that are calorie-dense and have a low nutrient density, which is generally accompanied by a decreased consumption of fruits and vegetables, being two of the primary sources of vitamins and minerals [2,9,37]. The NOVA food classification has established food processing as an important indicator of food quality. It divides foods into ultra-processed, processed, unprocessed, and culinary ingredients [38]. Ultra-processed foods (UPFs) account for more than 60% of the dietary energy intake and nearly 90% of added sugars in the diets of adults in the US [39]. UPFs are mainly ready-to-eat industrial formulations composed of processed ingredients refined from whole foods and usually have added fats, sugars, sodium, artificial flavors, colorings, and other food additives [40]. UPFs are nutrient-poor (low in dietary fiber, protein, micronutrients, and phytochemicals), energy-dense, and low-cost foods with important adverse health outcomes [39]. The obesity rate and its related nutritional deficiencies have been linked to an increased consumption of UPF, making children and adolescents their leading consumers [40,41]. In particular, sugar-sweetened beverages (SSBs) are strongly associated with weight gain and are recognized as a significant risk factor for type-2 diabetes, cardiovascular disease, and cancer. SSBs are one of the primary sources of added sugar in diets; a 355 mL serving of soda provides around 35–37 g of sugar and 140–150 calories [42]. Sweetened beverages are also recognized as nutrient-poor and linked to micronutrient deficiencies since their consumption is inversely correlated to the concentrations of vitamin D and calcium because of the lower intake of milk [2].

Another cause may be the higher nutrient requirements resulting from the pathophysiological and metabolic changes in individuals with obesity [2]. For example, obese patients present higher requirements of zinc, magnesium, chromium, manganese, and vanadium because they are involved in carbohydrate and fat metabolism. Thus, obese patients are at a greater risk of developing nutritional deficiencies related to these micronutrients [14].

Other studies have reported that increased adiposity and systemic obesity-related inflammation can disturb the absorption, distribution, metabolism, and elimination of micronutrients; obesity affects the protein binding, volume of distribution, hepatic metabolism, and renal clearance, mainly due to the elevated adiposity, blood composition and volume, cardiac output, lean body mass, and organ size (primarily liver and kidney) of obese patients [2,6,9]. For example, some minerals and lipophilic vitamins (vitamin D and A) can be sequestered in the adipose tissue, affecting their distribution, decreasing circulating concentrations, and reducing bioavailability for metabolically active tissues; obese people commonly have lower serum levels of vitamin D and A [6,11,14]. Obesity is also associated with deficiencies of water-soluble vitamins, including thiamine, folate, and ascorbic acid, partly because their excretion increases due to their high expenditure [14]. Elevated levels of triglycerides, cholesterol, and free fatty acids in the bloodstream of obese subjects may impact the distribution of protein-bound micronutrients. Likewise, minerals with chemical similarities to other compounds within the food matrix can compete for transport proteins or other absorption mechanisms, hindering their absorption and bioavailability [2,6].

Additionally, the treatment of morbid obesity involving bariatric surgery can increase the risk or aggravate micronutrient deficiencies by reducing their consumption or absorption [9,11]. Its effect and significance will depend on which part of the gastrointestinal tract is bypassed; for example, zinc, iron, manganese, selenium, chromium, calcium, and the vitamins A, C, E, K, folate, thiamine, biotin, riboflavin, niacin, pyridoxine, and pantothenate are absorbed in the duodenum and jejunum, whereas fat-soluble vitamins and vitamin C are absorbed in the ileum. In particular, vitamin B_12_ first binds to intrinsic factors in the stomach, and is then absorbed in the ileum [2,9]. Patients undergoing gastric bypass and related surgeries have a higher risk of presenting a malabsorption of micronutrients that are primarily metabolized and/or absorbed in the stomach and the first part of the ileum [2].

**Figure 1:**
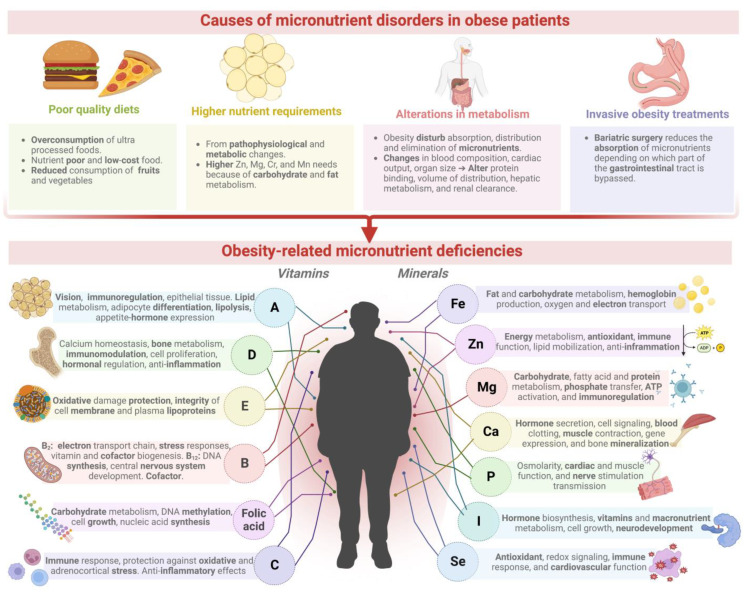
Micronutrient deficiencies associated with obese patients. Ca: calcium, Cr: chromium, Fe: iron, I: iodine, Mg: magnesium, Mn: manganese, P: phosphorus, Se: selenium, Zn: zinc. Figure created with BioRender.com.

Several studies have been performed to study deficiencies in micronutrients in individuals with obesity (Figure 1), Guan et al. [43] evaluated nutritional deficiencies in Chinese patients undergoing Roux-en-Y gastric bypass (RYGB) and sleeve gastrectomy (SG). They found several nutritional deficiencies in the bariatric candidates, identifying vitamin D deficiency as the most severe (78.8%), followed by vitamin B_1_ (39.2%), vitamin B_6_ (28.0%), folate (26.8%), vitamin C (18.0%), transferrin (11.6%), and phosphorus (11.5%). In a preoperative evaluation of 200 candidates for bariatric surgery, Pellegrini et al. [44] found that 85.5% of the patients presented at least one micronutrient deficiency: the most prevalent were vitamin D (74.5%), folate (33.5%), iron (32%), calcium (13%), vitamin B_12_ (10%), and albumin (5.5%). Similarly, Asghari et al. [45] studied the micronutrient status of morbidly obese candidates for bariatric surgery (mean age: 37.8 years, mean BMI: 44.8 kg/m^2^): deficiencies were identified for vitamin D (53.6%), vitamin B_12_ (34.4%), and serum iron (10.2%). In another study performed with 1732 patients with morbid obesity (age: 40 ± 12 years, mean BMI: 44 ± 9 kg/m^2^), data showed a high prevalence of micronutrient deficiencies: 63.2% of the patients presented deficiencies in folic acid (<5.3 ng/mL), 97.5% in vitamin D (<75 nmol/L), 9.6% in iron (ferritin < 15 μg/L), 6.2% in vitamin A (<1.05 μmol/L), and 5.1% in vitamin B_12_ (<188 pg/mL) [46]. McKay et al. [6] found associations between an increased BMI and low serum micronutrient levels in overweight and obese Australian adults (BMI: 25–40 Kg/m^2^, age:18–65 years) compared with the clinical micronutrient references. Significant associations were found for vitamin D (*p* = 0.044), folate (*p* = 0.025), magnesium (*p* = 0.010), and potassium (*p* = 0.023). Table 1 summarizes the most common micronutrient deficiencies observed in individuals with obesity.

In addition to obesity-related micronutrient deficiencies, several studies have demonstrated that most of the global population currently suffers from micronutrient insufficiencies [2]. According to the World Health Organization, the most common micronutrient deficiencies include zinc, iron, iodine, and vitamins A, D, and B_12_ [79]. Micronutrient deficiencies affect more than two million people worldwide: 60% of people are iron (Fe)-deficient, 30% are zinc (Zn)- and iodine-deficient, and 15% are selenium-deficient [23,24]. In addition, the World Health Organization reported that a third of humans had been affected by zinc deficiencies. It is also estimated that 50% of children do not obtain the vitamins and minerals necessary for their development [19]. Therefore, it is critical to take global actions and develop strategies to counteract and cover the gaps in micronutrient intake in individuals across all weight categories, particularly in individuals with obesity, who represent a severe health and socio-economic problem worldwide.

## 3. The Current Unsustainability of Agri-Food Systems Contributes to Micronutrient Malnutrition

Agriculture is the most important productive sector worldwide since it is the primary food source, playing a crucial role in human nutrition and health [18,19,80]. However, (1) the current massive increase in population, (2) the shifts in dietary patterns and their related growth in fodder use due to the increased meat consumption (considering that 61.1 kg of grain is required to produce 1 kg of beef protein, and 38 kg of grain for the production of 1 kg of pork protein), and (3) the intensification in biofuel production has driven a substantial global rise in the demand for crop production (Figure 1), estimating that, in order to feed a population of 10 billion people in 2050, it will be necessary to increase food production by 70%, which represents a large amount of pressure to the agricultural sector [15,16,18,26].

Therefore, current actions and regulations on food security are mainly focused on food quantity and daily calorie intake, with very little attention on the food quality and the effects of malnutrition [26,81]. Consequently, the conventional agricultural system is designed for intensive food production at the expense of producing increasingly nutrient-deficient foods and consuming excessive amounts of fertilizers, pesticides, energy (mostly from fossil fuels), and water, thus generating severe problems regarding public health, pollution, soil degradation and environmental damage on global agroecosystems [20,21,22,23,24,81].

Long-term cropping leads to the depletion of nutrient reserves and organic matter in the soil, decreasing soil fertility; annually, nitrogen, phosphorus, and sulfur deposits are reduced by 42% (1500 kg/ha), 27% (290 kg/ha), and 33% (150 kg/ha), respectively [19,82]. Thus, applying chemical fertilizers is now the standard measure for providing additional nutrients to the soil, supplying soils with nitrogen, phosphorus, and potassium as the three most relevant macronutrients for plant growth and development [26]. Synthetic chemical fertilizers are overused to sustain and increase crop productivity [17,20,21,83]. To date, approximately 200 million tons of fertilizers have been consumed globally [84], and plants only use between 20 and 50% of the applied fertilizer; there is a low nutrient assimilation efficiency and a high rate of fertilizer release into the environment [17,20,21,85]. Chemical fertilizers are used because they can meet the plant nutrient requirements within a short period and bring rapid results [83]. However, overfertilization is the leading cause of current agricultural unsustainability [21,86].

The Intergovernmental Science-Policy Platform on Biodiversity and Ecosystem Services (IPBES) reported that the rapid expansion of croplands and intensive cropping systems had made agriculture the primary driver of soil degradation [87,88]. It is estimated that 52% of agricultural land and 33% of the world’s land are moderately or severely degraded [89,90]. Soil degradation currently affects 40% of the world’s population and costs between 6.3 and 10.6 trillion US dollars annually, being one of the most significant environmental problems [87,89,90,91,92,93]. Modern agriculture enhances soil degradation by increasing erosion, compaction, salinization, acidification, and soil contamination [88,94]. An important parameter used to define soil quality is the soil enzymatic activity, which consists of accumulated enzymes that come from cells of microorganisms, plant and animal residues, and proliferating microorganisms and have an important role in releasing trace elements and nutrients, promoting soil processes such as organic matter disintegration, nitrogen fixation, and xenobiotics detox, maintaining soil fertility, and providing adequate conditions for plant growth. Soil enzymes and microbial activity are sensitive to changes in the soil pH, temperature, and humidity, which are parameters that are highly influenced by soil management (organic and inorganic fertilization, irrigation patterns, crop rotation), environmental pollutants, and climatic variations; harmful processes such as soil degradation may induce shifts in the soil biological activity, destabilize agroecosystems, and cause a reduction in soil nutrient assimilation and use [95]. Soil degradation threatens food security as it decreases food production by reducing soil fertility and productivity. It is estimated that 24 billion tons of fertile soil are lost each year, projecting that, in the following 25 years, land degradation will decrease worldwide food production by 12% and up to 50% in some regions, increasing food prices by 30% [87,89,91,96,97].

**Figure 2:**
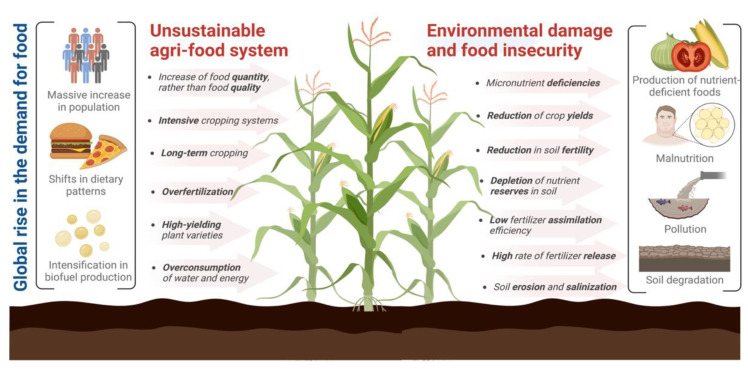
Unsustainable agri-food system contributing to food insecurity and environmental damage. Figure created with BioRender.com.

Modern agricultural practices, including crop intensification, large-scale irrigation, the use of high-yielding varieties, and fertilization focused on macronutrients (nitrogen, phosphorus, and potassium) with little or no use of micronutrients and organic components, have also contributed significantly to the alarming rates of micronutrient deficiencies worldwide. Deficiencies cause plant development alterations, eventually reducing the agricultural products’ nutritional value; even moderate nutritional deficiencies influence plant development [18,19,21,22,23,24]. For example, modern-day cereal varieties generally contain lower micronutrients such as zinc and iron [22,23]. In addition, it has been observed that, in rural communities whose diet mainly consists of agricultural products, there are recurrent health problems associated with micronutrient deficiencies in food [23,24]. Micronutrient deficiencies in crops are the main attribute for causing approximately 20% of death in children under the age of five, whose diets are generally deficient in essential nutrients, leading to malnutrition [24]. Providing nutritious, diverse, and balanced diets rich in macro and micronutrients is a big challenge for agriculture [17,98]. A more in-depth evaluation of the parameters determining the nutritional quality acceptance of agricultural products in food markets is required [26]. Figure 2 summarizes how the current unsustainable agri-food system contributes to the development of micronutrient deficiencies, food insecurity, and environmental damage.

Concerning obesity, identifying effective and safe strategies for long-term weight management is key to reducing the prevalence of overweight and obesity and mitigating obesity-associated health risks [99]. It is well known that the first-line treatment of obesity is dietary management. Dietary guidelines vary significantly in the optimal proportion of calories added by fat, carbohydrate, and protein; treatments for weight loss generally include “healthy” unprocessed foods, with a particular emphasis on reducing the intake of saturated fat and increasing the intake of fruit and vegetables, those being an important source of fiber and beneficial micronutrients [9,99]. However, based on the current unsustainable agri-food systems, agricultural products may be worsening the co-occurrence of undernourishment and obesity since foods may not be adequately providing the required amounts of macro and micronutrients, which are especially important when applying low-energy diets [9,13,26].

## 4. Potential Sustainable Agricultural Practices Capable of Fighting Obesity-Related Micronutrient Deficiencies

The diet of most of the world’s population depends on plant-based foods or derivatives of agricultural products that regularly have low concentrations of micronutrients and are unable to meet the Recommended Dietary Allowances (RDAs) [30]. The RDAs, which refer to the recommended daily intake levels of nutrients to meet the known nutrient requirements of practically all healthy individuals [100], are standard for minerals and vitamins but, based on scientific and clinical evidence, it seems to be necessary to develop specific adjustments for patients with obesity at its different levels [6,14].

Obesity-associated micronutrient malnutrition has received attention globally. Several ways to tackle micronutrient deficiencies include dietary variation, supplementation, industrial fortification, and biofortification [30,101,102,103,104]. Dietary variation or diversification is a food-based strategy that refers to the intake of various dietary sources, particularly plant-based foods (fruits, vegetables, and whole grains) [30,105]. The problem with this strategy is that foods containing high micronutrient levels are less accessible for rural communities because they are more expensive and scarcer, where their diets primarily consist of starch-based food. In addition, animal-based foods will be deficient if fodder is not enriched in micronutrients since animals rely on plant nutrients for nourishment [19]. Food supplements involve the consumption of micronutrients in pills, tablets, powders, or solutions when food cannot supply sufficient nutrients [106]. It has several disadvantages compared to other approaches to improve nutrition, including that it is not cost-effective for low-income consumers, can easily lead to overdosing, and may be unsustainable for large populations since it does not solve the root cause of micronutrient deficiencies [2,19,31]. Industrial fortification implies the addition of micronutrients to foods during the processing; for instance, vitamin D-enriched milk or iodized salt. However, fortified foods are mainly available and affordable for urban consumers [19,30,105].

Finally, the last approach used to improve the nutritional profile of plant-based foods is through biofortification. According to the World Health Organization (WHO), “biofortification is the process by which the nutritional quality of food crops is improved through agronomic practices, conventional plant breeding, or modern biotechnology” [104,107,108]. Biofortified crops have a higher content of macro and micronutrients in the edible parts of plants; they also ensure nutrient bioavailability to satisfy the daily nutrient demand [19,23,24,31,81,102]. Biofortification is considered a sustainable strategy intended to reach both the poor population and high-income people capable of addressing malnutrition and reducing micronutrient deficiencies in humans, especially since it represents an attractive approach to reducing nutrient inadequacies associated with obesity [19,22,23,31,107].

Biofortification through agronomic practices has received much attention because it involves different fertilization protocols with broad implications for environmental health. On the one hand, conventional mineral fertilization is associated with significant fertilizer losses due to leaching and volatilization, a low efficiency, and a severe environmental impact [19,105]. On the contrary, biofertilization, nanofertilization, and foliar fertilization practices are increasingly studied and implemented due to their efficiency, safety, reduced environmental impact, low cost, and ability to improve the utilization of chemical fertilizers and soil nutrients, while, at the same time, decreasing the consumption of conventional fertilizers [18,20,21]. Research on developing more sustainable agricultural practices to improve food security is of significant relevance. Therefore, we will discuss the use of biofertilizers, nanofertilizers, and foliar fertilizers, focusing on explaining their mechanisms of action and some important considerations for their formulation to understand how they increase the nutritional profile and productivity of agri-food relevant crops.

### 4.1. Biofertilization Strategies Based on Native Plant-Growth-Promoting Microorganisms

Biofertilizers are preparations of plant-growth-promoting (PGP) microorganisms that promote plant growth by influencing plant metabolism and improving the nutrient uptake from soil reserves [15,83]. Beneficial soil microorganisms colonize the rhizosphere and plant root system, exchange nutrients and signaling metabolites, and improve plant growth through various mechanisms, including mineral solubilization (phosphate, potassium, zinc), atmospheric nitrogen fixation, the production of phytohormones (auxins, cytokinins, gibberellins) and enzymes (phosphatases, catalases), heavy metal sequestering, the mineralization of soil organic matter, the secretion of siderophores, and the suppression of phytopathogens [15,17,20,21,30,32].

The use of biofertilizers has become vital because they can improve crop yields (10–40%) [109,110,111], enhance the micronutrient content [109,112,113], reduce chemical fertilization (35 to 50%) without compromising crops yield [114], and improve the plant resistance to stressful environmental conditions [115,116]. Soil microorganisms can improve the nutritional value of food by enhancing plant metabolism, soil micronutrient phytoavailability and uptake, the production of phenolic compounds and photosynthetic pigments, and antioxidant activity. The inoculation of various PGP bacteria, including *Azotobacter*, *Enterobacter*, *Pseudomonas*, and *Bacillus* species has been reported to improve the nutrients levels of economically important crops (Table 2) [17]. The biofertilization strategy is considered to be an economical and eco-friendly alternative to improve plant nutrition that positively impacts the environment and restores soil fertility [30].

Hussain et al. [117] evaluated an organic fertilizer based on a zinc oxide (ZnO)–orange peel waste composite enriched with Zn solubilizing bacteria (*Bacillus* sp. AZ6) in a 6:4 ratio in maize (*Zea mays* L.) under field conditions. The treatment significantly increased grain and shoot Zn (46% and 52%, respectively), crude protein (12.86%), fiber (2.87%), gluten (11.925%), and mineral (1.53%) contents compared to the control. In addition, the dry shoot-biomass, plant height, photosynthetic rate, transpiration rate, stomatal conductance, chlorophyll contents, and carotenoids were also increased by 46%, 53%, 53%, 42%, 45%, 57%, and 17%, respectively. Zaheer et al. [118] found that treating chickpea (*Cicer arietinum* L.) with two P-Zn-solubilizing bacterial strains (*Pseudomonas* sp. strain AZ5 and *Bacillus* sp. strain AZ17) enhanced the grain yield by up to 17% over the non-inoculated control. In particular, the *Pseudomonas* strain increased the Zn uptake, P uptake, grain yield, straw weight, nodules number, and nodule dry weight by 26.12%, 22.59%, 17.47%, 16.04%, 26.32%, and 22.53%, respectively, compared to the control. In another study, the nutritional quality of mungbean (*Vigna radiata*) was improved by applying iron-chelating rhizobacteria (*Pantoea dispersa* MPJ9 and *Pseudomonas putida* MPJ6). At harvest time, treated plants exhibited significant increases in vegetative parameters, iron content (3.4-fold increase), protein (2.5-fold increase), and carbohydrates (1.5-fold increase) compared to un-inoculated plants [111].

**Table 2:** Studies of micronutrient biofortification using beneficial soil microorganisms.

Targeted Plant	Evaluation	Improvement in Nutritional Value	Contribution to Crop Productivity	Reference
Maize (*Zea mays* L.)	Seed priming with *Alcaligenes* sp., *Bacillus* sp., *Pseudomonas* sp., and *Bacillus* sp.	Zn contents increased by 33.0%, 15.3%, 49.1%, and 15.6% in roots, grain, stem, and cob-pith.	Treatments improved cob length and diameter by 42% and 16.75%, respectively, and increased 100-grain weight by 18.4%.	[109]
Wheat (*Triticum aestivum* L. cv. Zhoumai)	Seed soaking and soil spraying with *Bacillus altitudinis* WR10 under field conditions.	Total N and K contents were enhanced by more than 50%. Fe content rose between 29.94% and 18.67%. Fe was accumulated mainly in the embryo and endosperm.	Inoculum increased kernels per spike (24.67–16.44%) and total chlorophyll content (42.07–22.85%).	[110]
Maize (*Zea mays* L.)	Field trial with *Bacillus* sp. AZ6.	Treatments improved grain Zn content (46%), shoot Zn content (52%), crude protein (12.8%), fiber (2.8%), carotenoids (17%), and chlorophyll content (57%), and decreased phytate (73%).	Biofertilizer increased plant height (10%-53%), dry shoot-biomass (46%), photosynthetic rate (47%), transpiration rate (42%), and stomatal conductance (45%).	[119]
Mungbean (*Vigna radiata* L.)	Pot study using *Pantoea dispersa* MPJ9 and *Pseudomonas putida* MPJ6.	Rhizobacteria showed iron-chelating activity (89.9–85.3%) and improved iron (3.4-fold), protein (2.5-fold), and carbohydrate content (1.5-fold).	Improved the maximum seed germination percentage (93.3%), shoot and root length, and fruit weight.	[111]
Coriander (*Coriandrum sativum* L.)	In vitro and greenhouse experiments with *Bacillus halotolerans.*	Increases in carbon (1.48%), calcium (1.23%), iron (179%), magnesium (3.30%), nitrogen (11.9%), and phosphorus (38.2%) contents.	Enhancement of stem length by up to 5.9%, shoot dry weights (15.8%), and chlorophyll content (34.1%).	[113]
Lettuce (*Lactuca sativa* L.)	Greenhouse test using *Pseudomonas* sp. and *Azospirillum brasilense* strains and nitrogen fertilizer doses (30–120 kg/ha).	Increases in carotenoid content (47%), ascorbic acid (42%), total phenolics (17%), and total chlorophyll (20%).	Bacteria improved plant height (15%), lettuce head fresh weight (48%), and root collar diameter (70%).	[112]
Cabbage *(Brassica oleracea* var. *capitata* L.)	Pot study with *Azotobacter* and phosphorus and potassium solubilizing bacteria.	Treatments increased vitamin C content by 17% and total soluble solids (TSS) of cabbage heads by 3%.	Improved the cabbage head’s polar diameter (8%) and equatorial diameter (4%).	[120]
Tomato (*Solanum lycopersicum* L. cv. Rio Grande)	Consortia of *Bacillus* species, *Azotobacter chroococcum*, and *Pseudomonas megaterium*.	Bacteria improved lycopene (52.8%) and total carotenoids (25%) contents, TSS, pectin methylesterase (PME), polygalacturonase (PG), and antioxidant (31.25%) activities in tomato fruit.	Increases in dry weight (39%), photosynthetic rate (9.9%), fruit weight per plant (26.78–30.70%), and yield (51.94%).	[121]

Even though biofertilizers represent a promising alternative to improve agricultural productivity, one of the main factors altering the biofertilizers’ efficiency is that most are produced with commercial strains that may not be adapted to adverse climatic conditions, hindering microbial colonization and survival [33,122,123]. Inoculation with native beneficial soil microorganisms is essential for the formulation and production of biofertilizers since these microorganisms have a greater adaptability and survival to edaphic and climatic conditions, increase the phytostimulation effects, and have shown a higher ability to increase crop yields, the plant stress-tolerance, and resistance against phytopathogens compared to inoculation with allochthonous strains. [34,123,124]. It has been observed that native PGP strains increase the effectiveness of microbial inocula, which may be due to coadaptation between local microorganisms and plant species [123]. Lauriano-Barajas and Vega-Frutis [33] highlighted that biofertilizer efficiency depends on the source of the microbial inoculum. They found that only native inoculants colonized plant roots, whereas commercial inocula had low or non-viable microbial propagules. Karnwal [125] evaluated the effects of native and commercial bacterial inocula on maize and wheat growth under saline conditions; the results revealed that native species led to the best growth parameters for both crops, including the shoot and root dry weight and length.

In addition, allochthonous inoculants can modify soil ecosystems by altering the metabolism of microbial communities. Changes in the structure of rhizospheric microbial communities could harm crop productivity if native symbiotic relationships are decreased or lost [33,122]. Armada et al. [122] investigated the effect of the inoculation of native PGP bacteria on the composition of soil microbial communities under drought conditions. The results indicated that native microorganisms do not have adverse effects on microbial populations. They improved the plant growth by increasing the nutrient uptake and assimilation and doubled the AMF colonization levels, concluding that inoculation with native species does not damage local communities and has positive effects on plant development in degraded soil.

Another important consideration in developing efficient biofertilizers is the use of microbial consortiums. They have been demonstrated to be more effective in increasing crop yields and plant growth than individual strains [17,116,124]. Konappa et al. [126] assessed the individual and combined inoculation of several rhizospheric microorganisms belonging to the genera *Trichoderma* (eight isolates), *Bacillus* (six isolates), *Pseudomonas* (three isolates), and *Brevibacillus* (one isolate) to determine their ability to protect tomato against bacterial wilt caused by *Ralstonia solanacearum*. They found potential benefits on the plant growth, pathogen suppression, and tomato yield of inoculating combination treatments of beneficial microorganisms compared with an individual application, possibly due to an additive and complementary effect between microbial species; thus, consortia effectiveness may depend on the synergistic interaction of their constituents [127]. 

Developing effective biofertilizers requires an exhaustive study of the interactions between microorganisms, plants, and their environment. Plant and microbial responses differ when exposed to diverse environmental conditions [33,34,128]. Biofertilization protocols should be designed for each specific environment considering the humidity, temperature, soil properties, and native microbial communities already colonizing the area [86,122,128].

#### 4.1.1. Direct and Indirect Plant-Growth-Promoting Mechanisms to Biofortify Food

**Figure 3:**
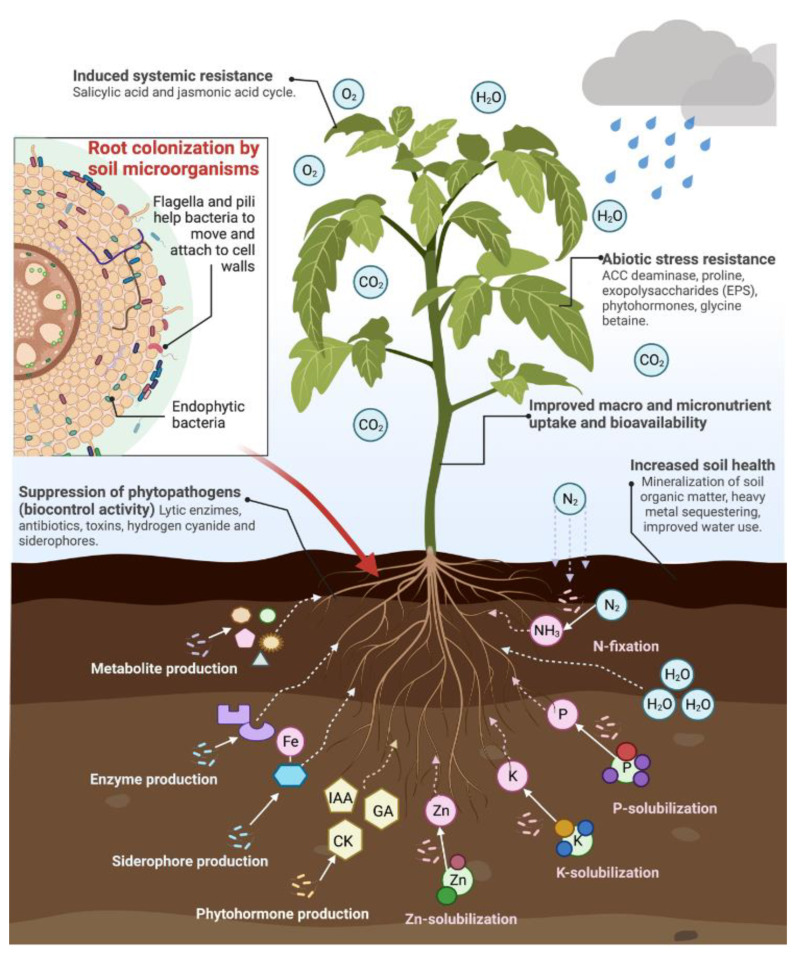
Plant growth and health-promoting mechanisms of soil microorganisms. Fe: iron, IAA: indole acetic acid, CK: cytokinin, GA: gibberellic acid, Zn: zinc, K: potassium, P: phosphorus, NH_3_: ammonia, N_2_: atmospheric nitrogen. Figure created with BioRender.com.

Beneficial microorganisms present several mechanisms through which they influence plant development, food quality, and soil health [17]. Mechanisms are classified into two main groups: direct and indirect plant-growth-promoting mechanisms. Direct plant growth promotion stimulates plants’ growth directly by providing nutrients and growth stimulators. It involves mechanisms such as nitrogen fixation, the solubilization of macro and micronutrients, and production of plant growth regulators (phytohormones) and enzymes. Indirect methods are mainly related to the biocontrol of pathogens through the synthesis of antibiotics, lytic enzymes, hydrogen cyanide, and siderophores. These compounds suppress phytopathogen growth by competing with pathogens for nutrients or releasing toxic chemicals (Figure 3) [15,129,130]. Next, some of the mechanisms that directly influence the micronutrient content of agricultural products will be described. These mechanisms can also improve the content of other biologically active compounds, such as phenolics, that can contribute to weight loss and reduce the incidence of metabolic complications associated with obesity [6].

##### Zinc Solubilization

Zinc (Zn) is the most important micronutrient needed for plants’ normal development and metabolism [109]. It plays a vital role in various physiological processes, including hormonal regulation, protein synthesis, carbohydrate and lipid metabolism, chlorophyll production, cell membrane integrity, gene regulation and expression, and functions as a cofactor of enzymes [22,23,108,131]. Zinc deficiency is one of the most recurrent problems decreasing crop productivity. It is estimated that 50% of the arable agricultural soil is deficient in soluble Zn, generating reduced crop yields and a poor nutritional quality of agricultural products [23,131]. In the soil, more than 84% of total Zn can be found as ZnO, ZnCO_3_, Zn_3_(PO_4_)_2_·4H_2_O, and ZnS, whereas only 1% is water-soluble and available for plant absorption (Zn^2+^). Therefore, Zn is integrated into chemical fertilizers, but most of this soluble Zn is rapidly fixed and converted to insoluble forms, and only 1–4% of the applied Zn can be used by plants [22,23,108,129,130,131,132].

Micronutrient bioavailability and distribution in soil depend on different physical, biological, and chemical parameters, such as soil texture (clay soil decreases the bioavailability of Zn), soil pH (low pH levels increase Zn solubility, whereas neutral or high soil pH levels inhibit Zn dissolution), ligand–metal complexation (PO_4_^3−^ and CO_3_^2−^ contents), organic matter levels (soils with a high organic matter increase micronutrients availability; organic matter increases solubility by providing chelating ligands that bind to micronutrients), cation-exchange capacity, dissolution, precipitation, and acid–base balance [108,132].

Some species belonging to the genera *Acinetobacter*, *Bacillus, Enterobacter*, *Pseudomonas*, *Serratia,* and *Xanthomonas* have been described to have zinc-solubilizing characteristics [133]. Species belonging to the genera *Bacillus* and *Pseudomonas* are of particular interest since they have been reported in different studies to have important implications for promoting plant growth and biofortifying food crops with zinc [129,133]. The primary mechanism by which microorganisms transform insoluble zinc sources into soluble, bioavailable Zn forms is through a reduction in the pH of the rhizospheric soil. Minor changes in the soil pH significantly impact the release of micronutrients in the soil. The Zn bioavailability increases a hundred times with a one-unit decrease in pH [119]. Plant-growth-promoting microorganisms produce organic acids (citric, formic, acetic, gluconic, lactic, malic, 2-keto gluconic, and oxalic acids) that acidify the rhizosphere and increase the mineral bioavailability [118,134].

##### Siderophores Production to Increase Iron Bioavailability

Iron is a key element in plant cell reactions, cellular respiration, chlorophyll production, photosynthesis, intermediary metabolism, the tricarboxylic acid (TCA) cycle, lipid metabolism, DNA stability/repair, and oxygen transport [23,108]. Both zinc and iron are essential components for enzymes such as glutamate dehydrogenase (GDH), catalase, and superoxide dismutase, which participate in synthesizing chlorophyll and phytohormones. However, iron in degraded and alkaline soils presents a low bioavailability, resulting in iron-deficient foods [19,135]. Iron has two common oxidation states, Fe^3+^ and Fe^2+^, and is mainly found complexed with silicon (Si), oxygen (O_2_), or sulfur (S) [23,108].

Soil microorganisms and plants increase the iron bioavailability by releasing siderophores such as bacillibactins, pyoverdines, and cephalosporins, which chelate insoluble iron [111,130]. In iron-limiting environments, microbial siderophores scavenge Fe^3+^ from the mineral phases and generate soluble Fe^3+^-siderophores complexes that are absorbed into plant cells via iron-regulated surface membrane receptor proteins. Bacterial siderophores improve iron contents in plants and increase plant growth by enhancing the iron bioavailability near roots [111]. Several authors have also reported siderophores as potential biocontrol agents since these iron-chelating compounds deprive plant pathogens of this important micronutrient [15,17,130].

##### Nitrogen Fixation

Nitrogen-fixing microorganisms can reduce the application of nitrogen fertilizers and the environmental impact surrounding their use, which is essential for sustainable agriculture [83]. Nitrogen is the most influential macronutrient in plant development. It is an integral part of proteins, nucleic acids, and other important organic compounds [17,19,86]. Although around 78% of the nitrogen (N_2_) is free in the atmosphere, plants cannot use it [85]. Nitrogen is added to fertilizers as urea, anhydrous ammonia, and urea–ammonium nitrates, but its absorption and use depend significantly on the characteristics of the soil and the fertilizer; N-fertilizers have a reported use efficiency of 47%, meaning that more than half of the fertilizer is not taken by plants [91]. Therefore, farmers apply an excess to avoid deficiencies and ensure that there is enough available N for the crop [19,20].

Biological nitrogen fixation (BNF) is when nitrogen-fixing microorganisms convert atmospheric nitrogen into plant-assimilable chemical forms using an enzymatic complex called nitrogenase [15,17,83]. There are two types of BNF: symbiotic nitrogen fixation, which involves members of the family Rhizobiaceae, and leguminous plants [136]. The legume–Rhizobium symbiosis develops from phenolic compounds secreted by plant root exudates, mainly flavonoids and isoflavonoids, which bind to the transcriptional regulator NodD and activate the transcription of nodulation factors (Nod factors). This signaling pathway triggers the synthesis of lipoquitooligosaccharides (LCOs), which are necessary for nodule formation and infection. Subsequently, bacteria colonize the legume roots and form an organelle called a symbiosome derived from plant membranes, where nitrogen fixation and nutrient exchange between symbionts take place [137,138]. For nitrogen fixation, nitrogen gas (N_2_) diffuses through the soil to the nodules, which is converted to ammonia (NH_3_) by the bacterial nitrogenase enzyme complex. NH_3_ can be incorporated into the amino acid synthesis via the glutamine synthetase–glutamate synthase (GS-GOGAT) pathway or transported outside the bacteria to the plant cytoplasm via ammonia transporters, where it is used to synthesize nitrogen compounds, including amino acids, proteins, and alkaloids, in exchange for microbial nutrient molecules (glucose, amino acids). To maintain NH_3_ flux out of the bacteria, bacterial nitrogen metabolism (amino acid biosynthesis) is directly altered by the plant to force nitrogen excretion [15,32,83,116,139].

The other type of BNF is nonsymbiotic nitrogen fixation, which involves species of different genera such as *Arthrobacter*, *Acetobacter*, *Clostridium*, *Azotobacter*, *Bacillus*, *Pseudomonas*, and *Diazotrophicus*, and non-legume plants [17,83]. In this process, the atmospheric nitrogen is also converted into ammonia by the bacterial nitrogenase enzymatic complex, and NH_3_ is transported outside the bacteria and then absorbed by plants [116,139].

##### Phosphorus Solubilization

P-solubilizing microorganisms (PSM) are also important in maintaining agricultural productivity since P is the second most vital macronutrient for optimal plant growth [17,83,86]. It is essential in almost all major metabolic pathways, including signal transduction, protein synthesis, cell division, tissue development, energy transfer, photosynthesis, macromolecular biosynthesis, and respiration [17,83]. Phosphorus deficiency is one of the most common limitations for crop growth. Although the soil has abundant phosphorus deposits (400–1200 mg/kg of soil), this mineral is mainly insoluble and not available for plant use; only 1–5% in the soil is bioavailable [19,20,83]. In addition, most of the phosphorus in chemical fertilizers is unavailable for plants as it rapidly fixes, forming insoluble complexes, hindering its absorption and assimilation; most phosphorus in the soils is attached to cations (Ca^+2^, Al^+3^, Fe^+3^), which makes it unavailable [17,129,130].

PSM is essential in producing soluble phosphate forms and increasing the mineral bioavailability. They act by lowering the pH of the environment by producing mineral-dissolving compounds, including organic acids (indole-3-acetic acid (IAA), gluconic acid, lactic acid, 2-ketogluconic acid, oxalic acid, acetic acid, and citric acid, with gluconic acid being the most frequent solubilizing agent), inorganic acids (such as sulfuric, nitric, and carbonic acids), hydroxyl ions, siderophores, protons, and carbon dioxide [32,83]. Organic acids have hydroxy and carboxy groups that chelate cations (Ca^+2^, Al^+3^, Fe^+3^) attached to phosphates and release P ions by substituting cations. Other alternatives are H^+^ proton release derived from NH_4_ assimilation or the solubilization of organic phosphate (mainly inositol phosphate (soil phytate)) by removing P with non-specific acid phosphatases (NSAPs) (mainly phosphomonoesterases, also known as phosphatases) and phytases that dephosphorylate organic compounds releasing P [17,83]. There are many bacterial genera (*Pseudomonas*, *Agrobacterium*, *Bacillus*, *Azotobacter*, *Burkholderia*, *Burkholderia*, *Serratia*, *Rhizobium*, and *Enterobacter*) and fungi (*Penicillium*, *Rhizopus*, *Cladosporium*, *Fusarium*, *Aspergillus*, and arbuscular mycorrhizal fungi) recognized as important phosphorus solubilizers [17,32,83,129,130].

##### Potassium Solubilization

Potassium is the third major nutrient for plants, important in reproduction, photosynthesis, enzyme activation, the development of root hairs, the growth of pollen tubes, water regulation, and tolerance to abiotic factors [17,19,83]. In addition, potassium is involved in the functioning of around 60 different enzyme systems in plants [83]. In the soil, the soluble potassium concentration is deficient; 90% of potassium is present as silicate minerals and insoluble rock [130]. Potassium deficiency has become an important constraint in crop productivity [83,86].

Potassium solubilization occurs similarly to phosphorus solubilization. Many bacteria (*Bacillus mucilaginosus*, *Bacillus circulans*, *Bacillus edaphicus*, *Burkholderia*, *Enterobacter hormaechei*, *Paenibacillus glucanolyticus*, *Paenibacillus mucilaginosus*, *Paenibacillus frequentans*, *Acidothiobacillus ferrooxidans*, *Arthrobacter*, and *Sphingomonas*) have been recognized as mineral potassium solubilizers. Approximately 90–98% of potassium is mineralized as feldspar (orthoclase and microcline) and mica (biotite and muscovite) [32,83,140]. Solubilization occurs by dissolving silicate minerals with microbial organic (oxalic acid, tartaric acids, 2-ketogluconic acid, succinic acid, citric acid, malic acid, gluconic acid, lactic acid, propionic acid, glycolic acid, malonic acid, fumaric acid, etc., the first five being the most important) and inorganic acids, chelation, acidolysis, and exchange reactions, where released H^+^ protons can directly dissolve the mineral potassium. The production of organic acids is the primary mechanism used by soil microorganisms to solubilize phosphorus and potassium [17,32,83,129,130].

#### 4.1.2. Nanofertilization as a Multifunctional Strategy to Biofortify Food Crops

The use of engineered nanoparticles (NPs) (less than 100 nm in size) has significant potential to improve modern agricultural practices, mainly due to the outstanding benefits of NPs on plant germination, nutrition, growth, and productivity [108,131,141]. Nanoparticles have a size between 1 and 100 nm, which gives them unique physicochemical properties with many advantages over their bulk macrostructures or ionic analogs, including a higher surface-area-to-volume ratio, electrical conductivity and mechanical strength, enhanced reactivity, and special functionalization properties, improving the plant nutrient assimilation, transport, and use [19,21,108,131,141,142]. The small size of NPs enables their passage through biological barriers, easily diffusing into the vascular system of plants after soil or foliar application [107,108]. Several studies have revealed that nanoscale micronutrients used as nanofertilizers present an increased plant nutrition and improved plant resistance against environmental stress, having a superior efficacy in comparison with conventional micronutrient fertilizers. Nanomaterials applied in agriculture increase the effectiveness of traditional products by between 20 and 30%; they have a higher absorption rate, utilization efficiency, and faster nutrient release. In addition, nanomaterials reduce the toxicity to the soil by increasing the nutrient availability, reducing chemical doses, and minimizing the adverse effects of overdosing [19,21,22,107,108,141,142,143].

On the other hand, NPs represent a new alternative for the biofortification of crops. It has been proven that plant nutrients can be enriched by applying nanoparticles [19,21]. Through nanofertilization, it is possible to increase the bioavailable mineral nutrient content in edible portions of food crops; metal- or metal-oxide-NPs can be employed to produce biofortified food crops to reduce obesity-related nutritional deficiencies [22,23,107]. Velázquez-Gamboa et al. [144] evaluated the biofortification of *Stevia rebaudiana* plants via nanofertilization with ZnO phytonanoparticles. NPs were applied to the roots at 75 mg/L concentrations. The results revealed that nanofertilization increased the zinc content by up to 406.8% over the control, and the total phenol (60.5%) and flavonoid (87.8%) contents were also improved without adverse effects on plant development or on the biosynthetic pathway of steviol glycosides, which are responsible for the sweetening power of *S. rebaudiana* and have also been reported with important antihyperglycemic and antihyperlipidemic properties. Yang et al. [143] also evaluated ZnO NPs on the rice (*Oryza sativa* L.) yield, nutrient absorption, grain nutritional quality, and Zn biofortification. The authors found that NPs increased the nitrogen, phosphorus, and potassium content in rice grains, the number of panicles (3.8–10.3%), spikelet number per panicle (2.2–4.7%), and total biomass (6.8–7.6%). Nanofertilization enhanced the Zn levels of brown rice by 13.5–39.4% compared with conventional fertilization with ZnSO_4_. Supplementing Zn in the form of a nanofertilizer is an attractive alternative to improve the bioavailability of this nutrient and enhance plant growth [131]. Zinc oxide nanoparticles (ZnO-NPs) are the most widely used metal oxide nanoparticles; they provide a soluble and bioavailable source of zinc [131,143].

Some other nutrients that directly or indirectly have been biofortified using nanofertilization in edible crops include phosphorus, potassium, iron, iodine, calcium, selenium, copper, zinc, boron, magnesium, vitamin C, vitamin E, and carotenoids (Table 3). Additionally, NPs can induce the biosynthesis of secondary metabolites of interest, promote the activity of some plant enzymes (e.g., phosphatase, amylase, nitrate reductase, and phytase) involved in nutrient acquisition and metabolism, stimulate the production of photosynthetic pigments and the photosynthesis process, improve the stomata opening and CO_2_ assimilation, and regulate oxidative stress by inducing enzymatic antioxidants such as superoxide dismutase, catalase, and peroxidases [102,107]. The relevance of biofortifying minerals is based on the fact that vitamins cannot be absorbed alone or work in the absence of minerals [105].

Interestingly, nanoparticles, as well as soil microorganisms, have also been demonstrated to biofortify plants with anti-obesogenic phytochemicals during their cultivation, representing a viable alternative to overproducing bioactive compounds such as polyphenols (phenolic acids, flavonoids, lignans, tannins), alkaloids, photosynthetic pigments, lipids, fibers, and proteins, which have shown anti-obesogenic potential in several plant species. Anti-obesogenic phytochemicals have revealed to manage obesity by suppressing appetite, reducing adipogenesis and lipogenesis, inhibiting digestive enzymes to reduce lipid and carbohydrate absorption, enhancing lipolysis and thermogenesis, regulating gut microbiota, and suppressing inflammation induced by obesity [145].

**Table 3:** Studies of nanobiofortification of agri-food crops using nanosized micronutrients.

Targeted Plant	Assay	Improvement in Nutritional Value	Contribution to Crop Productivity	Reference
Tomato (*Solanum lycopersicum* L.)	Greenhouse experiment applying copper nanoparticles (Cu-NPs).	Enhancement of potassium (16%), vitamin C (122%), lycopene (106%), total protein (99%), total phenols (36%), and flavonoids (16%) contents.	Tomato fruit firmness improved by 29%. Titratable acidity (TA) decreased by 16.33%. TSS increased by 6%.	[146]
Melon (*Cucumis melo* L.)	Shade house trail using Cu-NPs.	Cu-NPs increased copper content (540%), vitamin C (22%), phenolic (39%), and flavonoid (28%) contents in the melon pulp.	Fruit weight was increased by 41%, fruit firmness by 29%, and TSS content by 25%.	[147]
Bell pepper (*Capsicum annuum* L.)	Greenhouse test with selenium, silicon, and copper nanoparticles (Se-, Si-, and Cu-NPs) under saline stress.	Treatments improved 76% lycopene (76%), β-carotene (51%), phenols (65%), and flavonoid (175%) contents in fruit.	Chlorophyll a was increased by 79%, chlorophyll b by 75%, and total chlorophyll by 72–52%.	[148]
Tomato *(Solanum lycopersicum* L.)	Pot study evaluating Se-NPs.	Fruit magnesium, iron, zinc, and phenol increased by 29.8%, 27.6%, 21%, and 39%, respectively. Selenium was bioaccumulated in the fruits.	Shoot and fresh root biomass increased by 35% and 20.7%. Number of fruits and fruit postharvest longevity improved by 25.3% and 38%.	[149]
Mango *Mangifera indica* L. cv. Zebda and Ewasy	Field trial with 14-year-old mango trees using NPKMg nanoparticles.	NPs enhanced vitamin C (18%), total sugar (30%), and TSS (19%) in mango fruit. Leaf N, P, and K chlorophyll increased by 19%, 34%, 18%, and 26%, respectively.	Nanofertilizer increased the fruit edible portion (48%), fruit weight (28%), shoot length (23%), and yield per tree (47%).	[150]
Strawberry (*Fragaria ananassa*)	Field experiment with *Botrytis cinerea* infected plants applying calcium carbonate (CaCO_3_)-NPs and iron oxide (Fe_2_O_3_)-NPs.	Nano-treatment increased vitamin A (10.8-fold), C (1.7-fold), and E (2.7-fold) in fruit. Ca and Fe contents also increased by 102% and 157%.	NPs improved cell wall fractions such as cellulose (58.7%), pectin (108%), hemicellulose (131.7%), and lignin (1.61%) in fruits, and decreased *B. cinerea* infection by 85.6%.	[151]
Coriander *(Coriandrum sativum* L.)	Growth chamber assay with titanium dioxide nanoparticles (TiO_2_-NPs).	TiO_2_-NPs elevated Na (5%), K (26%), Ca (76%), Mg (67%), Fe (39%), Mn (107%), Zn (37%), and B (62%) in shoots. Soluble protein content increased by 21.1% in roots.	Shoot, and fresh root biomass (12.3% and 13.2%) and dry biomass (10.7% and 27.4%) were improved.	[152]
Wheat (*Triticum aestivum* L.)	Greenhouse test evaluating zinc and iron oxide nanoparticles.	Zn concentration improved in grain (105%), shoots (24%), and roots (19%). Fe concentrations also increased in grain (121%), shoot (28%), and roots (29%). Chlorophyll a (55%), chlorophyll b (133%), and carotenoids (112%) were also improved.	Plant height and spike length were increased by 37% and 50%. Shoot, root, spike, and grain (dry weights) were enhanced by 53%, 46%, 69%, and 74%. Cadmium contents decreased in grain, shoot, and root by 83%, 38%, and 55%.	[153]
Maize (*Zea mays*)	Field study testing ZnO-NPs.	ZnO-NPs enhanced N (78%), K (126%), P (20%), Zn (260%), and cellulose (8.5%) contents.	The number of plants (46%), plant height (15%), stover yield (40%) and fresh shoot (45%) and root (79%) weight were increased.	[154]

However, besides their positive impact on agriculture, nanoparticles can also adversely affect plants. Their beneficial effect or toxicity will depend on the route of exposure, dose, solubility, particle size and morphology, media composition, particle composition, and surface chemistry [107,131,155]. Previous reports have shown that NPs exert phytotoxic effects under certain conditions by inducing oxidative stress. Oxidative stress implies an imbalance between reactive oxygen species (ROS) production and the plant defense system [131], and can generate DNA damage, lipid peroxidation, protein oxidation, membrane damage, electrolyte leakage, and cell death. At specific dimensions, NPs can also block plants’ pores, interrupt the absorption of nutrients, alter germination, and damage chloroplasts that ultimately interrupt the photosynthesis process [21,107]. Small nanoparticles (<5 nm) can induce phytotoxicity even at low concentrations; small-sized NPs are easier to be absorbed and transported within the tissue and may have higher levels of accumulation and toxicity [156]. Other toxic effects on plants include a reduction in plant growth, shoot and root elongation, biomass production, and photosynthetic function [155]. In addition, the influence of NPs on soil microorganisms must be considered since NPs might also damage the microbial cell membrane and cell walls, affect cellular organelles, induce ROS production, and disrupt metabolism [155]. Therefore, the synthesis method, characterization, and evaluation of nanomaterials intended for agricultural use are essential for ensuring safe and effective agricultural products. 

#### 4.1.3. Importance of Nanoparticle Synthesis Method on Biofortification and Plant Growth Promotion

**Figure 4:**
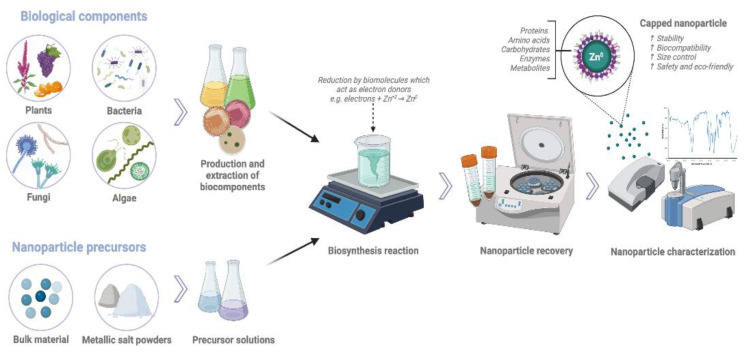
Biological or green synthesis methods for nanoparticle production. Figure created with BioRender.com.

There are different strategies used for synthesizing nanoparticles. Physical and chemical methods are more commonly used; however, they are time-intensive and costly. These methods also release toxic compounds that represent a risk to plants and the environment. Therefore, their biological applications are limited [156]. Biological or green synthesis methods have gained a lot of interest in recent years due to their biocompatibility, low toxicity, and ecological nature (Figure 4) [107]. Nanoparticle biosynthesis involves the use of biological substrates from microorganisms (e.g., bacteria, fungi, and algae), plants (i.e., roots, leaves, flowers, and seeds), and biomolecules (carbohydrates, enzymes, proteins) to carry out the nanoparticle reduction and stabilization processes. Plants biocomponents have functional groups that act as organic ligands that serve as electron donors and efficiently reduce precursors and metal oxides to create nanoparticles. In addition, nanoparticles are covered by those organic compounds (proteins, amino acids, functional groups, carbohydrates) that act as capping agents, increasing their stability and biocompatibility and decreasing the NPs’ toxicity. Biosynthesis employs environmentally friendly solvents, non-toxic chemicals, and renewable materials to produce nanoparticles. Therefore, it is considered a non-toxic, simple, efficient, safe, fast, and economically viable alternative [18,19,156,157].

Elemike et al. [19] reported that, when nanoparticles are used for agricultural purposes, it is advisable to use green synthesis methods for safety reasons. Biofabricated nanoparticles have a low toxicity and greater effectiveness and stability than chemically or physically synthesized nanoparticles. They improve plant growth promotion properties and stress tolerance and induce beneficial effects on soil microbial populations. Methods can be optimized by modifying the pH, temperature, and salt concentration conditions to control the nanoparticle size, shape, and dispersion [21].

##### Soil and Foliar-Applied Nanoparticle Absorption and Translocation through Plant Systems

Foliar fertilization is currently an important and highly efficient agricultural fertilization technique. It favors plants’ quick and effective assimilation of the applied nutrients, thus increasing crop yields and quality [158,159,160]. Foliar fertilizers are commonly used for the correction of nutrient deficiencies in crops, the maintenance of the plant nutrient status when environmental conditions reduce nutrient availability, and the supply of nutrients with a low phloem mobility (e.g., Zn, Fe, Mn, Ca, B) or in peaks of nutrient demand (e.g., flowering, fruit production) [161,162]. Foliar applications complement conventional fertilization practices, and, for some nutrients, soil parameters, plant species, and developmental stages, they could be more efficient and sustainable than soil fertilization [161,162,163]. In addition, foliar fertilization is characterized by the rapid absorption of nutrients; when nutrients are applied to the soil, the ability of the plants to use those nutrients lasts between 5 and 6 days.

On the other hand, foliar-applied nutrients are utilized within 3–4 days [159,161]. Foliar fertilizers deliver nutrients directly absorbed by leaves, which is also where photosynthesis occurs, so this involves the immediate availability of nutrients for plants [162,164]. In general, the foliar application of NPs has several advantages, including an improved uptake and assimilation of fertilizers, quick absorption by plants, controlled release of NPs, increased seed germination, reduced production of ROS, improved shelf life of agricultural products, and minimal impact on soil health. Spraying proper amounts of nutrients on crops foliage can lessen damage produced by traditional soil fertilization methods [156,165].

**Figure 5:**
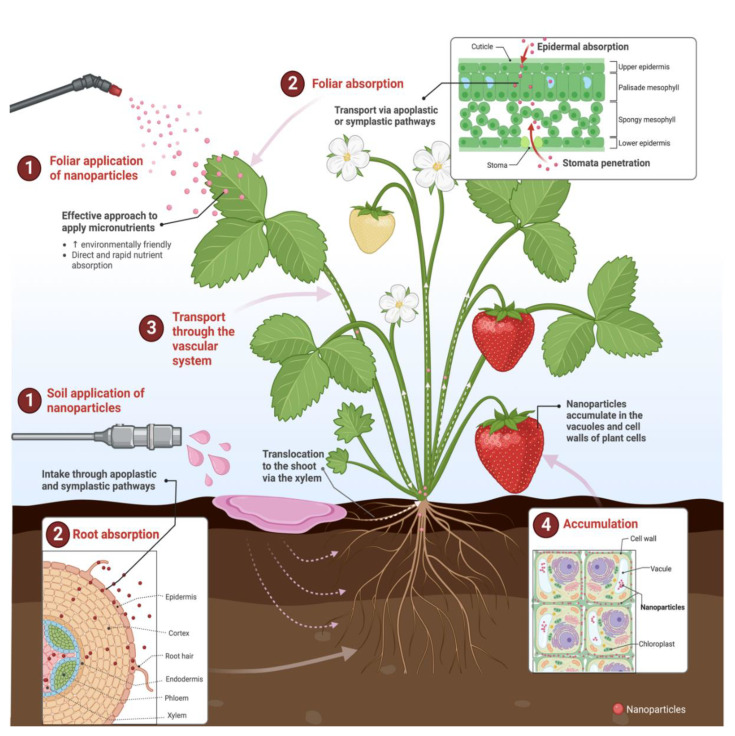
Soil and foliar nanoparticle uptake and transport through plant systems. Figure created with BioRender.com.

Foliar nanoparticles can be absorbed through stomata, water pores, carrier protein complexes, ion channels, trichomes, endocytosis, stigma, and wounds; the main absorption routes are stomata permeation and epidermal adsorption. Leaf pores have a 100 nm diameter, but waxy hydrophobic stomata have a smaller pore size, which can be blocked by large particles [156]. Reports have described that most inorganic metals enter plants through the stomata and leaf epidermis. Then, they are translocated via apoplastic or symplastic pathways (large particles (between 50–200 nm) are mainly transported via the apoplast, whereas small particles (10–50 nm) are generally transported through the symplast), and are finally transported to the roots and other plant parts through the vascular system (xylem and phloem) (negatively charged particles are more favorable for transport, whereas positive or neutral charge particles tend to accumulate on the plant vascular system) [19,21,135,156,166]. The vacuole and cell wall are important sites for the accumulation of NPs. For instance, metal-based NPs are sequestered in vacuoles by binding with compounds containing thiol groups (Figure 5) [156].

In the case of zinc, it is absorbed by epidermal cells, transported to the vascular bundle, and then translocated to the grain organ via the phloem. Iron NPs are more easily absorbed by the pores or foliar cracks of plants, and some chelating agents and enzymes promote iron transport within the plant [156,167]. The nanoparticle uptake and transport will depend on different factors, including th estomata distribution, growth stage, leaf surface area, and constitution of leaf veins, pores, and trichome density, among others. Large leaf surface areas, hard shoots, depressed leaf veins, and short petioles have been reported to accumulate more NPs [156]. Leaf hair and cuticular wax could interrupt nanoparticle absorption, and cell walls and waxes function as physical barriers to prevent the entry of foreign substances, including nanoparticles. In this sense, young leaves regularly have a higher capacity to absorb nutrients compared to mature leaves because new leaves have thinner wax layers and are less physiologically developed to block metal absorption [19,135,156,168]. The performance of foliar applications is also influenced by the characteristics of the liquid formulation (molecular size, pH, solubility, etc.) determining nutrient absorption and penetration, the environment (temperature, light, relative humidity) influencing the plant response, and the developmental stage and physiological status of the plant affecting the foliar treatment efficiency [158].

Regarding the absorption and translocation of soil-applied nanofertilizers, NPs interact with the root surfaces and root-damaged sites, cross through apoplastic and symplastic pathways within plant cells depending on the characteristics of the nanoparticles, and then move up to the shoot via xylem vessels, where they can be accumulated in the aerial plant organs (Figure 4) [19,21,131,135,156]. Soil-applied NPs provide several advantages for root cells, including an improved absorption of nutrients and increased disease resistance. However, roots absorb nanoparticles slower than plant stomata, and root exudates can inhibit the absorption of specific NPs. Therefore, the foliar application of nanofertilizers is more efficient in correcting nutrient deficiencies by providing nutrients directly to the leaves where they are required [156,169]. Foliar application can supplement special nutrient requirements without affecting the soil microbial environment since some NPs have been described as potent antimicrobials [156,170].

The absorption and transportation of nanoparticles will largely depend on the physicochemical properties of the soil (porosity, pH, salinity, organic matter) and the nanomaterial (concentration, size, and charge, which control agglomeration and aggregation) [21,171]. For example, the roots’ absorption of positive-charged metal-NPs is faster than negative-charged nanoparticles, which are more efficiently translocated to aerial plant organs [107].

##### Current Legislation and Regulations Regarding Food Biofortification

Many regulations and policies for ensuring food quality have been founded, but they mainly focus on food fortification. The European Union has a whole legislation addressed by the European Parliament to regulate food fortification. This regulation arranges ingredients and nutrients used in food manufacturing that may be added to fulfill standard requirements of those elements on the daily dietary intake [172]. Around fifty-four countries implemented mandatory fortification legislation between 2000 and 2020 [173]. However, only a few policies focused on biofortification have been established, which are mostly in low and middle-income countries. In 2020, the United Republic of Tanzania created the National Biofortification Guidelines to plan, implement, monitor, and evaluate biofortification initiatives. These guidelines center on producing specific biofortified crops rich in vitamin A, iron, zinc, lysine, and tryptophan by conventional plant breeding methods [174]. In Bangladesh, the National Strategy on Prevention and Control of Micronutrient Deficiencies proposed the use of a new variant of zinc-biofortified rice as a strategy to address micronutrient deficiencies [175].

The biofortification of nutrient-enriched staple crops in many countries around the world is reached by HarvestPlus, a program that is part of the CGIAR, a global agriculture research partnership for a food-secure future [176]. Through this program, until 2021, 283 varieties of 11 biofortified staple crops (e.g., iron bean, zinc rice, maize and wheat, and vitamin A orange sweet potato) were released in 30 countries across Asia, Africa, and Latin America [177]. According to the program, 24 countries have included biofortification in their policies and/or regulations. However, these are not clear and extensive, and, as described before, biofortification programs focus on staple crops [178]. In many countries, there is a lack of policies that guarantee crop quality. Most legislation focuses on processed food fortification, not the biofortification approach, mainly through fertilization practices such as bio- and nanofertilization protocols. It is crucial to develop and settle norms contributing to food quality to prevent and correct global health problems [26,98].

## 5. Conclusions

The information provided in this review is a robust and useful tool for understanding the prevalence of micronutrient deficiencies in obese patients, their leading causes and medical implications, how the current agricultural system contributes to increasing this problem, and how novel and sustainable technologies such as bio- and nanofertilization can be applied in agriculture to improve the nutritional quality of food to correct micronutrient deficiencies in obesity. We consider this review to be beneficial for future studies in the topic. Several reports have shown that poor nutrition in obese individuals contributes to the development of micronutrient deficiencies, which also contribute to the progression of obesity since these inadequacies affect the metabolism of carbohydrates and lipids. It was highlighted that unsustainable modern agricultural practices are primarily focused on the intensive production of food without caring about the quality of the products and the environmental impact generated by the overexploitation of natural resources. Incorporating plant-growth-promoting microorganisms as biofertilizers and nanoparticles as nanofertilizers has great potential to improve agricultural productivity and the nutritional value of agricultural products. In the case of biofertilizers, according to the literature, it is very important to include consortiums of native microorganism strains to increase their efficiency. There is a great potential to study and design safe and efficient bio- and nanofertilization products to enhance the content of deficient micronutrients in obesity and, in the same way, also quantify the effect on other compounds with anti-obesogenic activity to boost their effects against obesity.

Further research is needed to investigate the diversity, interactions, dynamics, and stability of native PGPM; the isolation and characterization of native soil microorganisms are crucial for finding potential candidates for the formulation of biofertilizers. Regarding nanofertilizers, various studies demonstrated the importance of applying nano nutrients to improve the food quality. However, more investigations are required to ensure safe and stable protocols for the ecological and scalable production of nanoparticles. The biofortification strategies are far from eradicating micronutrient deficiencies. However, they are a helpful tool that could complement other strategies and provide micronutrients to the population, contributing to the decrease in the progression of obesity and preventing obesity-related chronic diseases. An adequate intake of micronutrients is essential for the maintenance of health and the prevention of diseases.

## Figures and Tables

**Table 1:** Micronutrient deficiencies found in obese patients.

Micronutrient	Micronutrient Physiologic and Metabolic Function	Deficiency in Obese Patients	Type of Condition	Reference
Vitamin A and carotenoids	Retina and epithelial tissue development, lipid metabolism, immune system function. Inhibition of adipocyte differentiation by enhancing lipolysis. Reduction in leptin and resistin expression [14,47].	Carotenoids (α-carotene, β-carotene, ζ-carotene, lutein, and lycopene) ≈ 44.4%.	Male (n = 29) and female (n = 37) individuals between 49 and 58 years old with a body mass index (BMI) > 30 kg/m^2^.	[48]
		All evaluated patients presented a deficiency of vitamin A (<30 µg/dL).	Individuals with a BMI over 25 kg/m^2^ (overweight) and 30 kg/m^2^ (obesity) aged 18–65 years (n = 127).	[6]
Vitamin D	Calcium homeostasis, bone metabolism, immunomodulation, cell proliferation, and control of hormonal systems. Upregulates anti-inflammatory cytokines [49].	Approximately 16.5% presented a deficiency of serum 25 hydroxy vitamin D (<30 nmol/L).	Danish individuals; 6–18 years old (n = 1484) with overweight/obesity; body mass index standard deviation score (BMI Z-score) > 2.33.	[50]
		The prevalence of deficiency (≤20 ng/mL) is around 90%.	Obese individuals class II and III (BMI ≥ 35 and ≥40 kg/m^2^).	[2]
Vitamin E	Protection of cell constituents from oxidative damage, such as polyunsaturated fatty acids found in the membrane and plasma lipoproteins [51].	Deficiency of 61.5% (11.5 ± 12.2 mg/L), and 47.8% (15.6 ± 12.2 mg/L) in obese and metabolic syndrome patients, respectively.	Individuals 10–16 years old from Central Turkey with obesity (BMI Z-score > 2) (n = 73) or metabolic syndrome (waist circumference ≥ 90 cm (n = 64).	[52]
Vitamin B_2_	Mitochondrial electron transport chain function and homocysteine metabolism. Its derivatives, flavin mononucleotide and flavin adenine dinucleotide, are implicated in stress responses and vitamin and cofactor biogenesis [53].	Deficit of 48.9% in the obese group (89.1 ± 35 μg/L); 33.1% in the metabolic syndrome group (116.7 ± 65.2 μg/L).	Individuals 10–16 years old from Central Turkey with obesity (BMI Z-score > 2) (n = 73) or metabolic syndrome (waist circumference ≥ 90 cm (n = 64).	[52]
		Deficiency of 38.8% (<5 ng/mL).	Children 11–17 years old (n = 50) with obesity (BMI Z-score ≥ 2).	[54]
Vitamin B_12_	DNA synthesis, conversion of homocysteine to methionine, and central nervous system development. Cofactor in the one-carbon metabolism and propionate catabolism [55,56].	Insufficiency of 23% (< 150 pmol/l) in cohort 1 and 18.3% in cohort 2.	Two cohorts of pregnant women (16–18 weeks) (n = 244 and n = 60) with average BMI = 26.5 ± 5.5 kg/m^2^ for cohort 1 and BMI = 32.6 ± 11.2 kg/m^2^ for cohort 2.	[57]
		Deficiency of around 29% (397.5 ± 26.3 ng/L).	Forty obese adults (BMI > 35 kg/m^2^) aged 21–49 underwent bariatric surgery.	[58]
Folic acid	Well-functioning carbohydrate metabolism (15). DNA methylation, cell growth, and nucleic acid synthesis [56].	Prevalence of 54% (obese) and 65% (patients after bariatric surgery).	Patients with morbid obesity before (BMI > 30 kg/m^2^) and after bariatric surgery (BMI > 35 kg/m^2^).	[56]
		Inadequacies (<10 nmol/L) per area: America (0.8–2.1%), Europe and Eastern Mediterranean (40.9%), Africa (24.4%), Southeast Asia, and Western Pacific (1.1–3.7%).	Women with a rising prevalence of overweight and/or obesity (BMI > 18.5 kg/m^2^) in reproductive age (15–49 years old) in 17 population surveys.	[59]
Vitamin C	Immune response, protection against oxidative and adrenocortical stress. Anti-inflammatory effects [60].	Deficit of 24.6%, 32.8%, and 34.6% for sarcopenic, osteopenic, and osteosarcopenic obese individuals.	Korean women (n = 1344) postmenopausal (>50 years old) with osteosarcopenic (BMI = 27.15 kg/m^2^), sarcopenic (BMI = 28.12 kg/m^2^), and osteopenic (BMI = 26.24 kg/m^2^) obesity.	[61]
Iron	Fat and carbohydrate metabolism, hemoglobin production, oxygen transport, DNA synthesis, and electron transport [14,62].	Deficiency of 31.8% in male and 25.9% in female patients.	Children 8–9 years old (n = 160) with high body fat (BMI Z-score > 1) in Sri Lanka.	[63]
		Insufficiency in patients with peripheral (16.9%) and central (10.7%) adiposity.	Overweight and/or obese American young women (23–43 years old; BMI ≥ 25 kg/m^2^; n = 81).	[64]
Zinc	Energy metabolism with antioxidant and immunological properties. Stimulates the function of zinc-α2-glycoprotein (adipokine with lipid mobilizing and anti-inflammatory activity) [65].	Prevalence of 24–74% after bypass surgery: biliopancreatic bypass (45–91%), gastric bypass (15–21%), laparoscopic sleeve gastrectomy (11–14%).	Patients with morbid obesity before (BMI > 30 kg/m^2^) and after bariatric surgery (BMI > 35 kg/m^2^).	[56]
		Deficiency prevalence of 84.7% (<70 µg/dL fasted).	Women rising prevalence of overweight and/or obesity (BMI > 18.5 kg/m^2^) in reproductive age (15–49 years old).	[59]
Magnesium	Carbohydrate metabolism, phosphate transfer reactions, fatty acid and protein synthesis, ATP activation, and immune system function [62,66].	Deficiency in males was 6.6%, and, in females, was 7.7%.	Children 8–9 years old (n = 160) with high body fat (BMI Z-score > 1) in Sri Lanka.	[63]
Calcium	Hormone secretion, intracellular signaling, blood clotting, muscle contraction, gene expression, and bone mineralization [67,68].	Deficiency of 50.2% in obese women.	Obese women (35.37 ± 2.09 years old) with average BMI = 34.68 ± 0.61 kg/m^2^ (n = 70).	[69]
Potassium	Cellular osmolarity, acid–base equilibrium, cardiac and muscle function, and nerve stimulation transmission [70].	Deficiency of 59.6% in obese women.	Obese women (35.37 ± 2.09 years old) average BMI= 34.68 ± 0.61 kg/m^2^ (n = 70)	[69]
		100% of patients showed deficiency (<3.5 mmol/L).	Individuals with a BMI over 25 kg/m^2^ (overweight) and 30 kg/m^2^ (obesity) aged 18–65 years (n = 127).	[6]
Iodine	Thyroid hormones biosynthesis, vitamins, macronutrient metabolism, and cell growth fetal and child neurodevelopment [71,72].	Insufficiency prevalence of 24.4%.	Overweight (BMI > 25 kg/m^2^) and obese (BMI > 30 kg/m^2^) children (11–13 years old) residing in iodine-sufficient areas (IS) and mildly iodine-deficient areas (ID).	[73]
Selenium	Antioxidant defense, redox signaling, immune response, and cardiovascular function [74].	Deficiency of 25.9% in plasma and 34.2% in the erythrocyte.	Obese women aged 20–50 years (BMI ≥ 35 kg/m^2^, n = 63).	[75]
Copper	Electron transport, protein structure, mitochondrial respiratory chain, immune function, antioxidant defense. Cofactor of redox enzymes [56,76].	Concentration decreased by 16% 12 months after bariatric surgery.	Norwegian patients (85% women) 27–59 years old, eligible for bariatric surgery (BMI = 42.4 ± 3.6 kg/m^2^, n = 46).	[77]
		Prevalence of 46.7%.	Overweight/obese children aged 6–16 years (average BMI = 24.78 ± 3.93 kg/m^2^, n = 69).	[78]

## Data Availability

Not applicable.
